# Supplementary material for: Fundamental limits to learning closed-form mathematical models from data
Source: Nat Commun. 2023 Feb 24;14:1043. doi: 10.1038/s41467-023-36657-z (PMC9950473; doi:10.1038/s41467-023-36657-z)
Supplement: Supplementary file 1 — Supplementary information [file 41467_2023_36657_MOESM1_ESM.pdf]

SUPPLEMENTARY INFORMATION

Fundamental limits to learning closed-form mathematical models from data

Oscar Fajardo-Fontiveros,[1,*] Ignasi Reichardt,[1,2,*] Harry R. De Los Rios,[3] Jordi Duch,[3] Marta Sales-Pardo,[1,†] and Roger Guimerà[1,4,‡]

[1]*Department of Chemical Engineering, Universitat Rovira i Virgili, Tarragona 43007, Catalonia*
[2]*Department of Mechanical Engineering, Universitat Rovira i Virgili, Tarragona 43007, Catalonia*
[3]*Department of Computer Science and Mathematics, Universitat Rovira i Virgili, Tarragona 43007, Catalonia*
[4]*ICREA, Barcelona 08010, Catalonia*

LEARNABILITY TRANSITION FOR A GENERALIZED LINEAR ESTIMATION PROBLEM

In addition to the two models discussed in the body of the paper, we investigate what happens for a model not drawn from the prior $p(m)$, and with data generated differently from the two models in the main paper. In particular, we generate data using the generalized linear model $m^*(x, \theta^*) = |x \cdot \theta^*|$ with $\theta^* \in \mathbb{R}^d$, $x_i \in \mathbb{R}^d$, $d = 5$, $x_i \sim \mathcal{N}(0, I_d/d)$, weights $\theta^* \sim \mathcal{N}(0, I_d)$, and $N \in \{50, 600\}$. Individual, noisy observations are thus given by $y_i = |x_i \cdot \theta^*| + \epsilon_i$, with $\epsilon_i \sim \mathcal{N}(0, s_\epsilon)$.

As in the body of the paper, the question we are interested in is whether it is possible to identify the true generating model $m^* = |x \cdot \theta|$ (regardless of the precise value of the parameters), as opposed to competing models such as $m = (x \cdot \theta)^2$, $m = |x_1\theta_1 + x_2\theta_2 + \theta_3|$, $m = x \cdot \theta$, or any other expression. As we show in Fig. **??**, even though this model is not directly drawn from the prior and, in fact, has low a priori probability, and even though points are generated differently from the examples the body of the paper, the transition occurs exactly as in those examples and as predicted by the theory.

We also compare the predictive performance of probabilistic model selection to that of artificial neural networks (ANN) as in the main manuscript. This comparison is particularly interesting considering that the current generalized linear model should be exactly learnable by an ANN with only a few hidden units with ReLU activation functions. However, we still observe that the accuracy of the ANN on unobserved data is limited by the number of points in the low-noise regime. We argue that this is caused by *too much* expressiveness of the ANN. In the low-noise regime, the BMS is almost certain to identify the correct model, and thus it interpolates optimally between observations in the training set—only the correct model is considered. By contrast, the ANN has a lot of flexibility to interpolate, that is, it finds many acceptable ways to interpolate between the observed points. Thus, in this region, the accuracy of the ANN is not limited by the noise in the data but by the density of points in the training set—lower density means more possibilities to interpolate.

---

* These two authors contributed equally.

† Corresponding author: marta.sales@urv.cat

‡ Corresponding author: roger.guimera@urv.cat

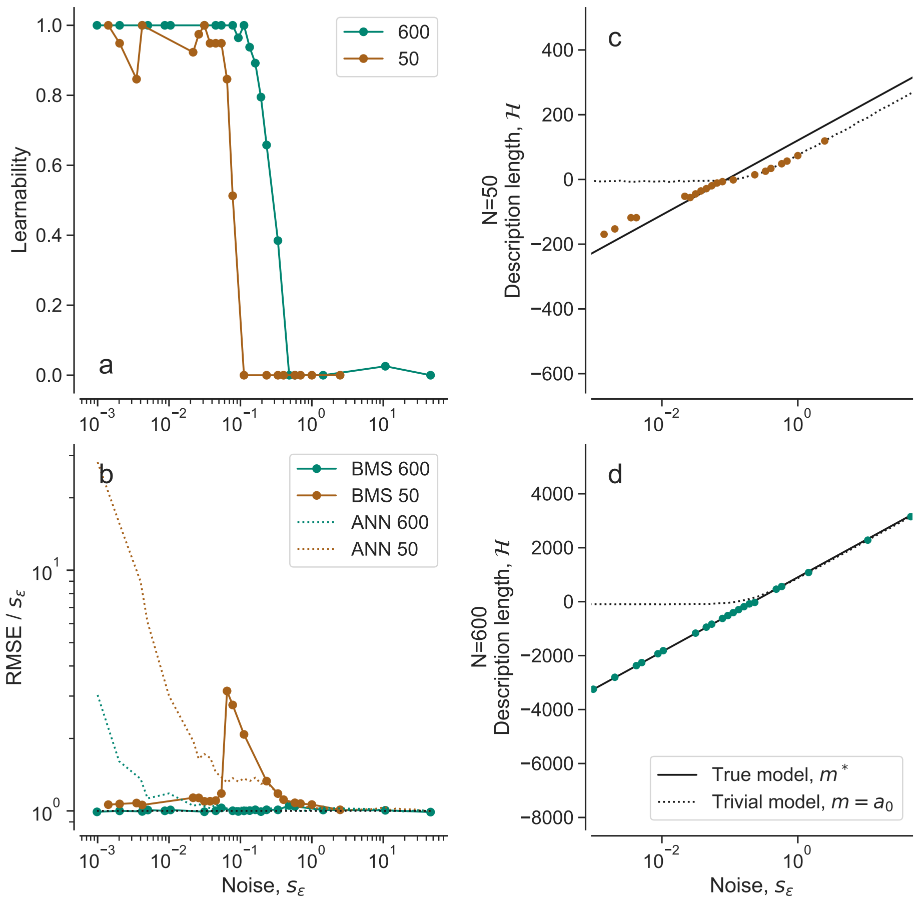

FIG. S1. **Learnability transition for a generalized linear estimation problem**. The true generating model is $y_i = |\theta^* \cdot x_i| + \epsilon_i$, with $\theta^* \in \mathbb{R}^d$ and $x_i \in \mathbb{R}^d$, $d = 5$. We generated data using $\epsilon_i \sim \mathcal{N}(0, s_\epsilon)$, $x_i \sim \mathcal{N}(0, I_d/d)$, weights $\theta^* \sim \mathcal{N}(0, I_d)$, and $N \in \{50, 600\}$. (a) Transition of the learnability parameter. (b) Prediction error to unseed data as a function of the observation noise s_ϵ, for probabilistic model selection and for an artificial neural network as in the main text. (c-d) We plot the description length of the MDL model identified by the Bayesian machine scientist, averaged over 40 realizations of the training dataset D (colored symbols). For each model and N, we also plot the theoretical description length of the true generating model m^* (solid black line) and of the trivial model m^c (dotted black line).
